# Circular RNA therapeutics: a new class of long-acting RNA medicines for oncology, immunology, and rare diseases

**DOI:** 10.3389/fimmu.2026.1758902

**Published:** 2026-04-07

**Authors:** Okechukwu Paul-Chima Ugwu, Michael Ben Okon, Fabian Chukwudi Ogenyi, Jovita Nnenna Ugwu, Chinyere Nneoma Ugwu

**Affiliations:** Department of Research Publication and Extension, Kampala International University, Ishaka-Bushenyi, Uganda

**Keywords:** cancer vaccines, circRNA, circular RNA, immunotherapy, lipid nanoparticles, rare diseases, RNA therapeutics

## Abstract

Circular RNAs (circRNAs) have progressed from being viewed as splicing by-products to emerging therapeutic constructs with a distinct pharmacology. Their covalently closed topology can increase RNA stability, prolong intracellular persistence, and under some conditions sustain translation relative to matched linear RNAs. However, circRNA performance and immunogenicity depend strongly on the circularisation chemistry, impurity profiles (linear RNA and double-stranded RNA (dsRNA) by-products), sequence and structural features, and the delivery formulation. Consequently, broad claims such as ‘circRNA is less immunogenic than mRNA’ are unreliable without rigorous, standardised benchmarking. This narrative review provides a conceptually grounded, evidence-informed synthesis of recent advances in synthetic (exogenous) circRNA therapeutics across oncology, immunology (including vaccines), and rare/chronic diseases. We combine structured literature identification (2018-November 2025) with qualitative appraisal of preclinical and early translational studies, focusing on: (i) platform engineering (circularisation, purification, translation elements, and delivery); (ii) therapeutic modality (protein-coding versus regulatory circRNAs and programmable circuits); (iii) disease-domain use cases; and (iv) unresolved controversies and translational constraints. We introduce a decision-oriented three-axis framework to delineate settings in which circRNA plausibly offers added value (for example, single-dose local protein depots; durable antigen expression for selected vaccine strategies) versus contexts where evidence remains preliminary or advantages may diminish when compared against optimised mRNA/self-amplifying RNA (saRNA) comparators. We also highlight emerging computational models that may accelerate circRNA target and drug-sensitivity discovery. Finally, we propose priorities for the field: standardised purity and identity assays, head-to-head platform comparisons, mechanistic immunoprofiling, and indication-focused early clinical development.

## Graphical Abstract

**Figure:**
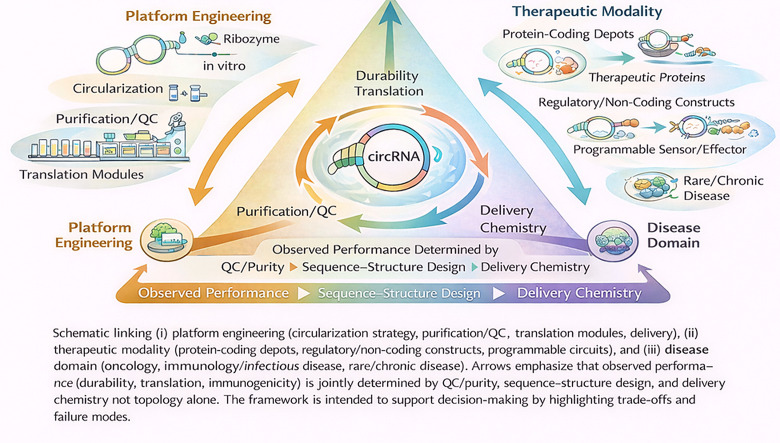

## 1 Introduction

The clinical impact of mRNA vaccines has catalysed renewed interest in RNA as a programmable therapeutic modality (1, 2). However, conventional linear RNAs can show limited intracellular stability, transient expression, and innate immune activation, which may complicate repeat dosing and reduce translation (5, 6). CircRNAs are covalently closed RNAs that lack free 5′ and 3′ ends; this topology can increase resistance to exonucleases and may alter RNA sensing and expression kinetics (7–10). Endogenous circRNAs are abundant across eukaryotic transcriptomes and contribute to diverse regulatory functions (1–4). Synthetic circRNAs exploit this topology to enable durable protein expression (for example, antigen or cytokine depots) or non-coding functions (for example, microRNA decoys) for therapeutic applications (10–12). Foundational studies reported that highly purified engineered circRNAs can sustain translation longer than matched linear RNAs *in vivo* and may attenuate certain innate sensing pathways. Subsequent work, however, indicated that impurities and RNA structural features can reverse this phenotype and provoke strong immunostimulation (13–17). CircRNA should therefore not be treated as intrinsically ‘immune-silent’; rather, immunogenicity is designable and quality-dependent (18–20). This review synthesises the current landscape of circRNA therapeutics in oncology, immunology, and rare/chronic diseases, with an emphasis on critical appraisal: (i) when reported advantages are robust and when they may not hold; (ii) where mechanistic interpretations diverge; and (iii) what evidence is required for credible clinical translation.

## 2 Conceptual framework

To organise heterogeneous evidence without repeating platform claims across sections, we use a three-axis framework:

1. Platform engineering: circularisation chemistry, purification/analytics, translation elements, and delivery.
2. Therapeutic modality: protein-coding depots versus regulatory/non-coding functions versus programmable circuits.
3. Disease domain: oncology, immunology/infectious disease, and rare/chronic diseases.

The framework is decision-oriented and focuses on trade-offs, failure modes, and benchmarking criteria that determine whether circRNA provides incremental or transformative benefit for a given indication.

**Table 1** summarises the principal design levers spanning circularisation strategy, purification and analytics, translation-control elements, and delivery systems, drawing on foundational studies in synthetic circRNA engineering and analytical methodology (7, 8, 13, 36, 40, 46, 50). It also highlights common failure modes yield limitations; junction-associated ‘scar’ sequences; concatemer or linear carryover; dsRNA by-products; assay under-detection; context-dependent translation variability; and delivery-driven inflammatory signals consistent with published concerns regarding circRNA immunogenicity and innate sensing, impurity generation and removal, and measurement and identification challenges (14, 21, 41, 49, 52), as well as efforts to develop ‘scarless’ circularisation systems (61, 66).

**Table 1:** Engineering choices, trade-offs, and failure modes.

Engineering layer	What is being optimised	Why it matters	Key trade-offs/failure modes	Practical decision cues
Circularisation strategy (e.g., PIE/self-splicing; ligation; scarless systems)	Yield, scalability, minimisation of junction ‘scars’	Influences purity, junction sequence, and dsRNA by-products	Low yield; junction ‘scars’; concatemer/linear carryover; dsRNA formation	Prefer approaches with demonstrated high purity and scalable QC; prioritise scarless designs if innate sensing is dose-limiting (7, 8, 13, 36)
Purification & analytics (HPLC/SEC/CE; dsRNA assays)	Removal of linear RNA/dsRNA; confirmation of topology	Immunogenicity and translation are often impurity-driven	Under-reporting of impurity levels; batch variability	Require dsRNA quantification (e.g., J2 antibody), linear RNA quantification, and topology assays before interpreting immunogenicity (40, 46, 50)
Translation control (IRES, m6A motifs, UTR-like modules)	Expression level, tissue bias, kinetics	CircRNAs are cap-independent; translation can be limiting	Viral IRESs may add unwanted motifs/structure; tissue variability	Select translation modules validated in the target tissue; avoid highly structured regions that generate dsRNA-like motifs (41, 49, 52)
Delivery (LNPs, polymers, exosomes; targeting ligands)	Biodistribution, endosomal escape, tolerability	Delivery often dominates PK/PD more than topology	LNP inflammatory signals; liver tropism; redosing constraints	Match delivery to indication (local depots versus systemic); minimise innate activation for chronic dosing (14, 21, 41, 49, 52)

**Table 2** contrasts areas with relatively mature preclinical validation (e.g., improved circularisation and translation frameworks and extended *in vivo* expression) (7, 8, 36, 46) with evidence gaps that limit generalisable claims, particularly the lack of standardised impurity and quality-control reporting and unresolved delivery-reactogenicity trade-offs (21, 49, 52). It delineates contexts in which circRNA is more or less likely to be competitive relative to optimised modified mRNA (modRNA), self-amplifying RNA (saRNA), or DNA/adeno-associated virus (AAV) approaches, informed by established principles of mRNA delivery and innate immune control (17, 20, 82, 84) and saRNA-specific innate/reactogenicity constraints and mitigation strategies (81, 83, 93).

**Table 2:** Mapping maturity and evidence strength across axes.

Axis	Most mature evidence	Evidence gaps that limit claims	Where circRNA likely ‘wins’	Where circRNA may not ‘win’
Platform engineering	Durable expression shown in small animals using purified constructs	Few standardised QC metrics; limited cross-laboratory reproducibility	When sustained expression is required and dosing frequency must be reduced	If optimised modRNA/saRNA achieves comparable duration with simpler CMC (7, 8, 36, 46)
Therapeutic modality	Vaccines and cytokine/protein depots in preclinical models	Regulatory/non-coding functions require stronger delivery and off-target analysis	Local protein depots; settings needing ‘programmable but non-integrating’ expression	Broad systemic protein replacement without long-term safety data (21, 49, 52).
Disease domain	Oncology vaccines and immune modulation (preclinical); infectious disease vaccine models	Chronic dosing in rare diseases; paediatric use; long-term safety	High unmet need with clear mechanism and measurable biomarker endpoints	Indications requiring lifelong stable expression (AAV/gene editing may dominate) (17, 20, 82, 84)

## 3 Methods

### 3.1 Review design and rationale

This article is a narrative review using a structured, transparent approach to identify and synthesise contemporary evidence on synthetic/engineered circRNA therapeutics. We selected a narrative design because the field remains methodologically heterogeneous across circularisation chemistries, translation-enhancement strategies, immunogenicity optimisation, and delivery platforms, making interpretive synthesis more appropriate than meta-analysis at this stage.

### 3.2 Aim and review questions

We aimed to: (i) map the therapeutic landscape of engineered circRNAs; (ii) summarise enabling platform technologies (circularisation, translation optimisation, delivery); and (iii) critically appraise the strength and limitations of preclinical claims supporting clinical translation.

### 3.3 Information sources and search strategy

We searched PubMed/MEDLINE, Web of Science, Scopus, Google Scholar, ScienceDirect, and Wiley Online Library for studies published from January 2018 to November 2025. Search terms combined controlled vocabulary (where available) and free-text keywords covering engineered circRNAs and therapeutic uses. Core concepts included:

- (‘circular RNA’ OR ‘circRNA’) AND (therapeutic OR vaccine OR protein expression OR drug)
- ‘synthetic circRNA’ OR ‘engineered circular RNA’
- ‘circRNA vaccine’ OR ‘circRNA cancer vaccine’
- ‘circRNA delivery’ OR ‘circRNA lipid nanoparticle’
- ‘circRNA rare disease’ OR ‘circRNA wound healing’ OR ‘circRNA sepsis’

We also screened the reference lists of influential primary studies and reviews to identify additional eligible papers.

### 3.4 Eligibility criteria

#### 3.4.1 Inclusion criteria

We prioritised peer-reviewed original research articles in English evaluating exogenous synthetic/engineered circRNAs as: (i) vaccines or protein-coding/protein-replacement therapeutics; (ii) immunomodulatory agents, gene regulators, or microRNA decoys; or (iii) enabling technologies supporting therapeutic deployment (circularisation methods, translation enhancement, delivery, immunological optimisation). Eligible domains included oncology, infectious disease/immunology, rare/chronic diseases, and mechanistic/translational studies.

#### 3.4.2 Exclusion criteria

We excluded studies restricted to endogenous circRNAs used solely as biomarkers without therapeutic intent; non-RNA circular constructs (e.g., circular DNA plasmids); and editorials/perspectives/commentaries lacking new experimental data (although we used selected pieces sparingly for conceptual framing).

### 3.5 Study selection process

We screened titles and abstracts for relevance, followed by full-text assessment. We prioritised mechanistically informative preclinical studies, translational work, and studies directly comparing circRNA approaches with established nucleic-acid modalities such as mRNA. After de-duplication and relevance screening, the final corpus comprised >50 primary studies plus a curated set of reviews supporting background context and field framing.

### 3.6 Data charting and synthesis approach

We charted and synthesised evidence thematically into three analytic strata aligned with the review aims:

1. Protein-coding circRNA therapeutics (vaccines; protein replacement; acute inflammatory, wound-healing, and sepsis models)
2. Non-coding/regulatory circRNA therapeutics (microRNA decoys; modulators of immunotherapy resistance; synthetic regulatory constructs)
3. Platform/methodology studies (circularisation strategies; translation-efficiency optimisation; immunogenicity profiling; delivery systems including LNPs and targeted nanoparticles)

### 3.7 Appraisal of study quality and risk of bias

Because most eligible evidence was preclinical, we performed a qualitative appraisal focusing on: (i) model choice and disease relevance (including *in vivo* validation); (ii) appropriateness of comparator groups, particularly circRNA versus mRNA benchmarking; (iii) analytical characterisation of circRNA preparations (e.g., circularisation efficiency and contamination by linear RNA/by-products); and (iv) experimental rigour (randomisation, blinding, sample-size justification, and statistical reporting).

Across studies, recurrent limitations included small sample sizes, short follow-up, heterogeneous reporting of immunogenicity/toxicity, and variable circRNA preparation quality. We used these issues to calibrate the strength of inferences throughout the synthesis.

### 3.8 Reporting framework

To align with SANRA expectations for narrative reviews, we: (i) stated a clear rationale and aims; (ii) described databases, time window, and representative search concepts; (iii) applied explicit eligibility criteria; (iv) organised the synthesis using a transparent thematic framework; and (v) integrated a structured qualitative appraisal to distinguish robust findings from provisional claims.

## 4 Platform engineering axis

### 4.1 Biogenesis and circularisation strategies

Synthetic circRNAs are produced by enzymatic ligation or self-splicing architectures (e.g., permuted intron-exon (PIE)). Recent ‘scarless’ and optimised chimeric designs aim to increase yield and reduce immunostimulatory by-products (21–23). Circularisation is not merely a manufacturing choice: it determines junction sequence/structure and impurity spectra (notably residual linear RNA and dsRNA), thereby modulating innate sensing and translation (24–26).

#### 4.1.1 Evidence-quality caveat

Comparative immunogenicity claims are frequently difficult to interpret because many reports omit quantitative batch QC. Without measured linear RNA and dsRNA burdens, statements such as ‘circRNA is less immunogenic’ remain underdetermined (27–30).

### 4.2 Translation control in circRNAs

Because circRNAs lack a 5′ cap, translation must be enabled by internal ribosome entry sites (IRESs), m6A-dependent initiation, or engineered internal translation modules (31, 32). Viral IRESs (e.g., EMCV) can drive robust expression but may introduce structured motifs that complicate innate sensing (33–35). Synthetic IRES/UTR-like elements can improve performance and may support tissue selectivity while reducing unwanted motifs; however, cross-tissue validation remains inconsistent (36–40).

### 4.3 Delivery systems: LNPs, polymers, and exosomes

LNPs remain the dominant delivery platform, drawing on established mRNA delivery strategies. However, delivery vehicles can be intrinsically pro-inflammatory; immunogenicity differences should therefore not be attributed to RNA topology without appropriate controls (41–43). Exosome-based approaches have also been explored for organelle targeting and immunomodulation, including in sepsis models (44–48).

### 4.4 Computational and AI-enriched circRNA discovery/design

Computational models are increasingly used in two complementary modes: (i) circRNA-disease association prediction to prioritise targets when experimental screening is limiting (49–52), and (ii) circRNA–drug sensitivity association prediction to generate hypotheses linking circRNA states to pharmacological response (53–57).

#### 4.4.1 Translational implication

These tools may shorten discovery timelines (target selection, biomarker nomination, resistance mechanisms), but they also raise the validation bar. Predictions should be anchored to clinically relevant datasets and tested using orthogonal experimental approaches to mitigate overfitting and false positives (58–62).

## 5 Therapeutic modality axis

### 5.1 Protein-coding circRNAs as ‘programmable depots’

The strongest translational rationale for circRNA is not simply ‘more stable RNA’, but longer-acting, programmable, non-integrating intracellular protein production a potential middle ground between repeated biologic injections and permanent gene transfer (63–65).

Where evidence is strongest: local or semi-local depots (e.g., wound bed applications and intramuscular vaccines) where prolonged expression after a single administration could reduce dosing burden (66–69).

Where evidence is weaker: broad systemic protein replacement requiring long-term repeat dosing, paediatric use, and comprehensive chronic safety datasets (70–72).

### 5.2 Non-coding and regulatory circRNAs

Non-coding circRNAs can act as microRNA decoys or protein-binding scaffolds, with potential to reprogramme oncogenic or immune pathways. Regulatory circRNAs, however, face additional hurdles: targeted delivery to the relevant cell states, off-target network effects, and challenges in defining dose–response relationships for network-level rewiring (73–75).

### 5.3 Programmable circuits and logic-gated circRNAs

Logic-gated circRNA circuits (e.g., microRNA-responsive switches, aptamer-controlled translation) offer conceptual precision but remain early-stage. Current evidence includes limited *in vivo* validation and an underdeveloped safety framework for unintended activation (76–79).

## 6 Disease domain axis

### 6.1 Oncology

Preclinical studies suggest that circRNA vaccines can elicit T-cell responses and may synergise with checkpoint inhibition in selected models. However, many oncology datasets remain small, tumour models are limited, and claims of ‘outperformance versus mRNA’ are often reported without fully optimised comparator conditions (dose, modifications, and formulation). The most plausible near-term use cases include settings with: (i) defined antigen targets; (ii) measurable immune correlates; and (iii) combination regimens where durability offers practical benefit (e.g., fewer boosters) (80–83).

### 6.2 Immunology and infectious disease

CircRNA vaccines have been explored for SARS-CoV-2 and other pathogens, with reports of improved thermostability and longer antigen expression. Superiority claims should be supported by head-to-head studies using optimised modRNA/saRNA comparators and standardised innate immune profiling, because delivery chemistry and dsRNA contamination can dominate outcomes (84).

### 6.3 Rare and chronic diseases

CircRNA therapeutics are conceptually attractive for rare/chronic diseases because they may offer longer-acting yet adjustable expression. However, this domain warrants particular restraint: chronic repeat dosing raises immunogenicity and safety questions that remain unresolved (85).

### 6.4 Recent breakthroughs and paradigm shifts

1. Durable *in vivo* translation is purity- and design-dependent. Engineered circRNAs can sustain translation and attenuate innate sensing when produced at high purity (87), but these advantages may diminish with residual linear RNA/dsRNA or innate-stimulatory junction/structure, reframing circRNA as an engineering space in which topology can help but QC and sequence architecture determine phenotype (88).
2. Vaccines show promise, but platform superiority remains unresolved. CircRNA vaccines in oncology and infectious disease elicit strong preclinical immunogenicity and, in some studies, more durable responses than linear RNA comparators (including SARS-CoV-2 variant models). However, inconsistent comparator optimisation and non-standardised assays limit definitive platform-level conclusions (89).
3. Protein-depot therapeutics clarify the value proposition. Non-vaccine circRNAs can function as local protein depots enabling single-dose or reduced-frequency dosing, exemplified by VEGF-A circRNA in diabetic wound healing (89) and circRNA-NGF neuroprotection in ocular injury models (90).

**Table 3** summarises representative circRNA vaccine applications across infectious disease and oncology by indication, antigen payload, and primary *in vivo* model (37, 75, 77, 79, 86), including optimisation studies targeting improved anti-tumour performance (31). It also identifies key uncertainties that limit the generalisability of current claims: (i) the need for head-to-head comparisons against optimised modRNA/saRNA benchmarks; (ii) standardised reporting of dsRNA and linear RNA impurities; (iii) consistent definitions of durability endpoints; and (iv) clearer evidence for transportability across tumour models and the feasibility of personalised manufacturing (21, 29, 52, 72–74).

**Table 3:** circRNA vaccines what they show and what remains uncertain.

Indication	Payload	Model	What looks promising	Key uncertainties/what to demand next
SARS-CoV-2/variants	Spike/RBD circRNAs	Mouse/NHP in key studies	Neutralising antibodies and T cell responses; potential durability	Require optimised modRNA/saRNA comparators; standardised dsRNA/linear impurity reporting; durability metrics defined consistently (37, 75, 77, 79, 86)
Cancer neoantigen/shared antigens	Tumour antigens	Syngeneic mouse tumours	CD8^+^ responses; potential synergy with immune checkpoint inhibitors (ICI)	Tumour model generalisability; manufacturing timelines for personalisation; immune escape and heterogeneity (21, 29, 52, 72–74)

**Table 4** compiles illustrative non-vaccine circRNA concepts and highlights settings in which prolonged ‘depot-like’ expression is mechanistically plausible, consistent with sustained-expression therapeutic demonstrations in regenerative and tissue-protective contexts (3, 4, 6) and related non-vaccine delivery concepts (5). Translational considerations include route-dependent risk, repeat-dose tolerability and immunogenicity, and scalable chemistry, manufacturing and controls (CMC) with standardised quality-control metrics including quantification of linear RNA and dsRNA impurities before efficacy claims are advanced (21, 41, 49, 52).

**Table 4:** Non-vaccine circRNA therapeutics with clearer ‘added value’ logic.

Indication	Payload	Delivery/route	Evidence signal	Translational feasibility considerations
Diabetic wound healing	VEGF-A circRNA	LNP, local application	Single-dose local expression; improved healing versus linear mRNA/protein in study	Local delivery may reduce systemic risk; still requires repeat-dose tolerability and QC standards (21, 41)
Diabetic wound healing	FGF2 circRNA	LNP, local	Sustained expression; improved closure and tissue regeneration	Similar local-depot rationale; requires long-term safety and scalable CMC (49, 52).
Ocular neuroprotection	NGF circRNA	LNP, intravitreal	Prolonged expression; retinal ganglion cell (RGC) protection; no overt retinal toxicity reported	Ocular route is relatively contained; candidate for early clinical translation if QC and immunology are acceptable (4, 6)
Sepsis immunomodulation	circRNA mSCAR (non-coding)	Exosomes + mitochondrial targeting	Improved survival; macrophage polarisation effects	Complex manufacturing; targeting reproducibility; safety/off-target concerns (3, 5)

**Table 5** contrasts circRNA, modified mRNA (modRNA), self-amplifying RNA (saRNA), and DNA/AAV by summarising strengths, best-fit use cases, and dominant constraints shaping feasibility and risk. It is anchored in literature on mRNA delivery, clinical translation, and innate immune mechanisms (17, 20, 82, 84), and on saRNA innate/reactogenicity and early clinical safety and immunogenicity evidence (81, 93). It situates circRNA opportunities and limitations within broader RNA-therapeutics framing and circRNA-specific technical constraints (e.g., impurity and quality control, and identification and delivery challenges) (29, 52).

**Table 5:** Platform comparison as a use-case guide.

Platform	Potential strengths	Where it is likely best	Major caveats
circRNA	Longer intracellular persistence; programmable depots; tunable immunogenicity	Local depots; booster-friendly vaccine concepts if purity/structure are controlled	Strong dependence on purification/QC; translation constrained by IRES/module choice; limited human data (17, 20)
modRNA	High expression; mature manufacturing ecosystem	Vaccines and many transient protein-expression uses	Duration may be shorter; innate sensing remains relevant; redosing constraints persist (82, 84)
saRNA	Intracellular amplification enables lower doses	Dose-sparing settings	Replicon-driven innate activation; less predictable kinetics (81, 93).
DNA/AAV	Long expression	Indications requiring long-term stable expression	Nuclear delivery/integration considerations (DNA); durability-reversibility trade-off and anti-vector immunity limiting redosing (AAV) (29, 52).

### 6.5 Current challenges, controversies, and knowledge gaps

#### 6.5.1 The ‘paradox’ of circRNA immunogenicity

CircRNAs are variably described as immune-evasive or strongly immunostimulatory (and sometimes exploited as self-adjuvants). This apparent paradox largely resolves when circular topology is separated from impurity-, structure-, and delivery-driven sensing (91).

##### 6.5.1.1 What actually triggers innate sensing?

Innate activation is most consistently explained by four non-mutually exclusive contributors: (i) dsRNA by-products from *in vitro* transcription/circularisation that engage MDA5/PKR/OAS–RNase L and endosomal TLR3; (ii) linear RNA carryover (including precursors/partial products) with immunostimulatory ends or chemistries and distinct trafficking; (iii) intrinsic sequence/structure (stable hairpins, duplex-forming/repetitive regions, certain viral IRESs) that increases dsRNA-like character after uptake; and (iv) delivery-driven inflammation, in which LNP composition/ionisable lipid chemistry and endosomal stress amplify cytokine signalling that may be misattributed to RNA topology (92).

Mechanistic takeaway: circRNA ‘immunogenicity’ is an emergent phenotype shaped by impurity burden, structural and motif content, and carrier chemistry, rather than a single intrinsic property of circular topology.

##### 6.5.1.2 Minimising unwanted immune activation: actionable design/QC rules

For chronic dosing or protein replacement, mitigation is primarily an engineering and analytics problem: quantify and reduce dsRNA (e.g., J2-based assays; chromatographic purification; optimisation of *in vitro* transcription), minimise linear contaminants and confirm topology (high-resolution separation plus nuclease-based validation), design against duplex formation (avoid long perfect complementarities and overly stable hairpins), and select translation modules with a low innate ‘cost’ (prioritising tissue-validated synthetic elements over highly structured viral IRESs where feasible) (93). Vaccine and protein-depot objectives differ: vaccines may tolerate or require controlled innate activation, whereas depots are often dose-limited by it, implying distinct QC thresholds and design priorities.

##### 6.5.1.3 Why some vaccine studies ‘benefit’ from immunogenicity

In vaccines, moderate innate signalling can function as an adjuvant and enhance priming. The field should, however, distinguish tunable adjuvanticity from impurity-driven inflammation, which can impair tolerability, reduce translation, and compromise repeat dosing (94).

## 7 Translational and clinical considerations

Near-term clinical translation of circRNA will likely be gated by: CMC/QC standardisation (junction identity, topology/circularisation efficiency, quantification of linear RNA and dsRNA, and batch consistency); rigorous benchmarking against matched, optimised comparators (modRNA/saRNA) using harmonised endpoints (expression kinetics, innate profiling, durability); delivery constraints (tissue targeting, repeat-dosing tolerability, lipid-associated inflammation); and the safety implications of prolonged expression (chronic overexpression, cryptic open reading frame translation, immune memory). These considerations make localised protein depots (wound and ocular indications) plausible first-wave use cases (90–93), while oncology adjuncts (local cytokine depots; vaccine checkpoint combinations) may be feasible where unmet need and immune readouts justify higher risk, provided QC and dosing strategies are mature (94–96).

### 7.1 Immune outcomes framework (circRNA vs modRNA/saRNA)

Immune outcomes depend on antigen-expression kinetics and the associated innate signals. circRNA durability is most useful when expression occurs in professional antigen-presenting cells (APCs) (80). This pattern can sustain antigen presentation and germinal-centre (GC) activity. Expression confined to non-APC parenchyma may increase antigen load without adequate costimulation (81). This imbalance may increase the risk of tolerance or dysfunctional T-cell states when innate cues are weak or persistent. LNP–modified mRNA (modRNA) typically produces rapid, high-peak expression. Its innate profile is shaped mainly by formulation, impurities, and nucleoside modification (82). Self-amplifying RNA (saRNA) can extend expression via replication. It is also more prone to amplified sensing and interferon-mediated translational shutdown. For circRNA, the key challenge is the ‘immunogenicity paradox’ (83). Highly purified constructs may be immunologically ‘quiet’. This may reduce reactogenicity but limit intrinsic adjuvanticity for robust T follicular helper (Tfh)/GC responses. By contrast, residual linear RNA or double-stranded RNA (dsRNA) can drive type I interferon (IFN) and interferon-stimulated gene (ISG) programmes (84). These programmes can suppress translation and skew differentiation. A useful cross-platform benchmark is antigen exposure relative to innate activation. This can be reported as antigen area under the curve (AUC) versus IFN/ISG AUC (85).

### 7.2 Redosing/boosting

Repeat dosing is limited by anti-delivery immunity, innate memory effects, and antigen-specific immunity. Anti-delivery immunity includes anti-PEG responses where relevant, and complement or excipient reactivity (80). Innate memory includes PRR desensitisation or trained inflammatory responsiveness with elevated baseline ISGs (81). Antigen-specific immunity can alter antigen handling and may promote dysfunctional states when antigen persists or dosing is too frequent. Redose regimens should prioritise formulation switching (82). They should also prioritise route switching and interval optimisation aligned with GC kinetics. Pharmacological innate modulation should be reserved for narrowly justified settings. Heterologous boosting (modRNA↔circRNA or saRNA↔circRNA) may reduce repeated exposure to identical excipients. It may also help tune the kinetics–innate activation balance (83).

### 7.3 Standardised immunoprofiling panel

Comparative studies should report a minimal, time-resolved immunoprofiling set. Early systemic mediators should include IFN-α/β (or validated surrogates), IL-6, TNF, IL-1β, CXCL10, and CCL2. A compact interferon transcriptional module should include ISG15, IFIT1, IFITM3, MX1, OAS1, and RSAD2 (84). These genes should be summarised as an IFN/ISG composite score. Mechanistic pathway readouts should include pIRF3, pSTAT1, and TBK1. Sensor-specific *in vitro* attribution should be provided where feasible. Immune readouts should be co-reported with RNA quality-control (QC) metrics (85). At minimum, include dsRNA burden, residual linear RNA fraction, and endotoxin/contaminants. APC phenotyping should capture maturation and trafficking (86). Report CD80, CD86, CD40, MHC-II, and CCR7 across relevant APC subsets. Adaptive correlates should include binding and neutralising antibodies. Include GC/Tfh readouts where feasible. Include antigen-specific CD4/CD8 responses by AIM, ICS, or ELISpot, with memory subset distribution (87). Translational safety signals should be reported in parallel. These include CRP where applicable, ALT/AST for systemic dosing, and complement markers when formulation reactions are plausible (88).

### 7.4 Safety, prolonged expression risks and de-risking

Prolonged expression can extend antigen presentation and support affinity maturation. It can also increase risk when it sustains inflammation or antigen presentation without resolution. Frequent boosting may further bias responses towards tolerance in low-danger contexts. It may also promote dysfunctional or exhaustion-like T-cell programmes under chronic antigen exposure with inflammatory cues. Duration should therefore be treated as a tunable parameter. Engineered circRNAs should minimise cryptic open reading frames (ORFs) and off-target translation (89–93). Risk mitigation should include in silico ORF scanning and empirical proteomics in relevant cells. Junction design choices should be reported transparently. Excessive innate activation is often impurity-driven rather than platform-intrinsic (94–96). De-risking should therefore prioritise orthogonal impurity assays and lot-to-lot QC thresholds. It should also correlate impurity metrics with IFN/ISG outputs. Delivery-associated hypersensitivity should be addressed separately. Monitor anti-excipient antibodies, complement activation, and infusion-like reactions where relevant (91). Use excipient minimisation and formulation or route switching, with prespecified monitoring plans. Long-term follow-up should consider high-risk subgroups, including autoimmunity-prone, chronically inflamed, or immunosuppressed populations (92).

**Figure 1:**
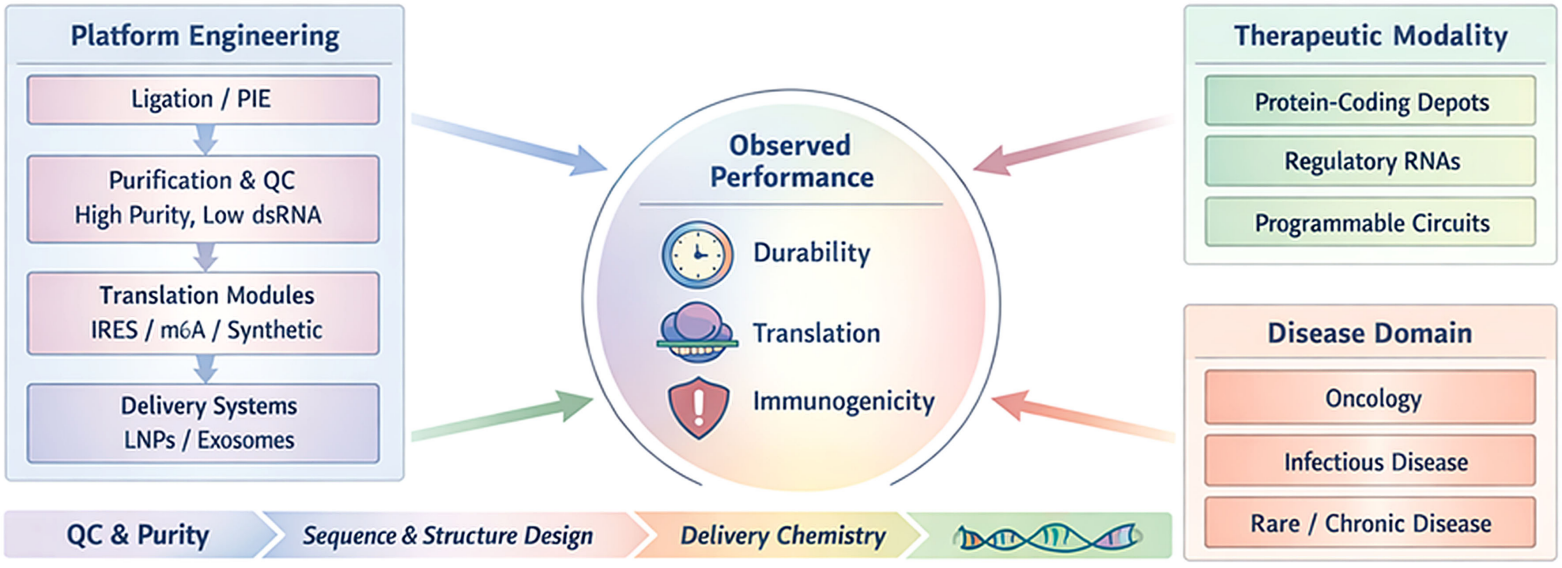
Integrated framework linking circRNA platform engineering, therapeutic modality, and disease domain to observed *in vivo* performance.

Platform engineering variables including circularisation strategy (ligation or PIE ribozyme), purification and quality control (QC; high purity, low dsRNA), translation module design (e.g., IRES, m6A motifs, or synthetic elements), and delivery systems (LNPs or exosomes) interact with therapeutic modality (protein-coding depots, regulatory RNAs, programmable circuits) and disease domain (oncology, infectious disease, rare/chronic disease). These inputs converge to determine observed performance, summarised as durability, translation, and immunogenicity. Key cross-cutting levers include QC/purity, sequence/structure design, and delivery chemistry (**Figure 1**).

**Figure 2:**
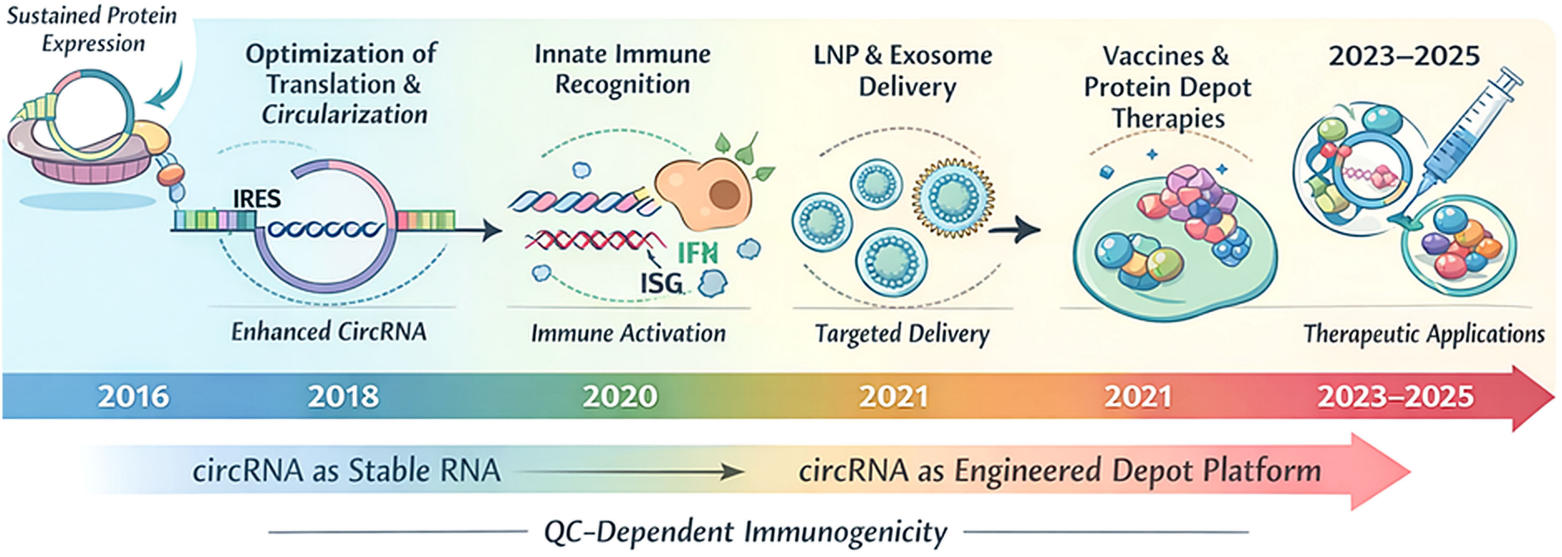
Milestones in the development of circular RNA (circRNA) therapeutics from stability-enabled expression to engineered depot platforms (2016-2025).

The schematic summarises key advances over time, beginning with recognition of circRNA as a stable RNA format supporting sustained protein expression (2016). Subsequent efforts focused on optimising translation and circularisation (e.g., IRES incorporation and improved circularisation strategies; ~2018), followed by increased understanding of innate immune recognition pathways, including interferon (IFN) and interferon-stimulated gene (ISG) responses (~2020). Delivery advances particularly LNPs and exosome-based approaches-enabled targeted *in vivo* delivery (~2021). These developments supported vaccine concepts and protein-depot therapies (~2021), culminating in broader therapeutic applications across 2023-2025. The lower band highlights the shift from ‘circRNA as stable RNA’ to ‘circRNA as an engineered depot platform’, alongside the persistent influence of QC on immunogenicity across stages (**Figure 2**).

**Figure 3:**
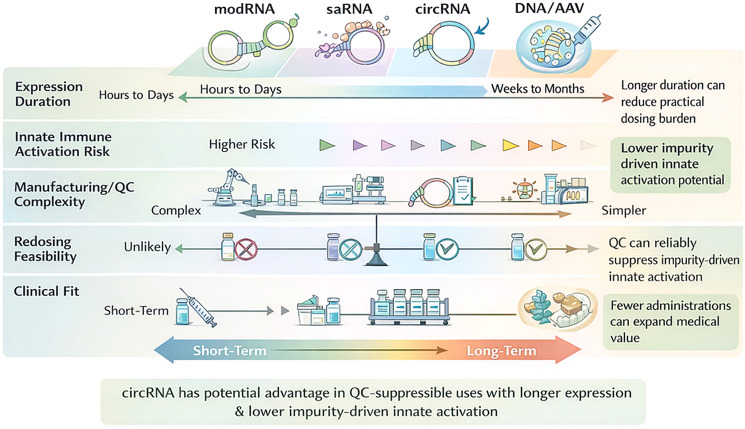
Comparative positioning of modRNA, saRNA, circRNA, and DNA/AAV platforms across key development and clinical-use attributes.

The schematic compares four nucleic acid delivery platforms with respect to: (i) expression duration (hours–days for modRNA and saRNA versus weeks–months for circRNA and DNA/AAV, depending on construct, formulation, and target tissue); (ii) risk of innate immune activation (influenced by sequence features and impurity profiles); (iii) manufacturing and QC complexity (including removal and monitoring of process- and product-related impurities); (iv) redosing feasibility (often more tractable for RNA modalities and constrained for viral vectors by anti-vector immunity); and (v) clinical fit along a short- to long-term treatment continuum. The figure emphasises that circRNA may be most compelling where longer expression can reduce dosing frequency and robust QC mitigates impurity-associated innate immune activation, improving tolerability and deployment (**Figure 3**).

## 8 Conclusion

CircRNA therapeutics represent a credible next-generation RNA modality, particularly as programmable, non-integrating depots for longer-acting protein expression and selected vaccine strategies. However, circRNA is neither intrinsically immune-silent nor universally superior to linear RNA platforms. Reported advantages often depend on circularisation chemistry, impurity control, sequence structure design, and delivery formulation. Priority needs include standardised QC/analytics, rigorous head-to-head comparisons with optimised modRNA/saRNA, mechanistic immunoprofiling to separate topology from impurities and delivery effects, and indication-focused translational programmes. If these priorities are addressed, circRNA could expand the RNA medicine toolkit, especially for local depots and selected immunotherapy/vaccine applications; broader systemic and chronic indications will require substantially stronger evidence for long-term safety and redosing.
